# Dual Language Competencies of Turkish–German Children Growing Up in Germany: Factors Supportive of Functioning Dual Language Development

**DOI:** 10.3389/fpsyg.2018.02261

**Published:** 2018-12-03

**Authors:** Beyhan Ertanir, Jens Kratzmann, Maren Frank, Samuel Jahreiss, Steffi Sachse

**Affiliations:** ^1^Department of Developmental Psychology, Institute of Psychology, Heidelberg University of Education, Heidelberg, Germany; ^2^Department of Psychology, Heidelberg University, Heidelberg, Germany; ^3^Department of Early Childhood Education, Catholic University of Eichstaett-Ingolstadt, Eichstaett, Germany

**Keywords:** bilingualism, language proficiency, dual language learners, second language skills, L1 skills, home language, L2 skills

## Abstract

This paper is about the first (L1) and second language (L2) skills of Turkish–German dual language learners (DLLs), the interrelatedness of the L1 and L2 skills, and their relation to other selected child and family variables. The first aim of the study was to examine L1 and L2 performance and the relation between the languages. Second, the study sought to explore the conditions in which functioning dual language development can be achieved, while trying to predict the extent to which child and environmental factors are related to the DLLs’ language competencies. L1 and L2 language skills of *N* = 69 bilingually developing 3–5 years old Turkish–German children were assessed via standardized tests. In addition, information on the children’s sociodemographic variables and home language environments was obtained by means of parental questionnaires. Correlational analyses were used to examine the interrelations between L1 and L2 skills and multiple regression analyses were conducted in order to predict the children’s language competencies. The children showed age-appropriate language skills in L1 (Turkish) and lower language skills in L2 (German). Whereas their phonological memory abilities were positively correlated with L1 and L2 skills, their expressive vocabulary in L1 was negatively correlated with L2 skills. Our findings also indicated that phonological memory was a strong predictor of language abilities. Concerning family variables, both early daycare entry and stimulating home language environment were significant predictors of better L2 skills. Lastly, balanced use of both languages at home had no negative consequences on language competencies. Although more research is needed, this study shows the benefits of using a combined language measure including both L1 and L2 skills to predict DLLs’ language competencies without disregarding either of their languages.

## Introduction

The population of children who speak Turkish as their first language makes up the largest group of dual language learners (DLLs) living in Germany (Autorengruppe Bildungsberichterstattung, 2016). Surprisingly, Turkish language is not losing its importance in Turkish–German families, even though most of them are now in their third or fourth generation as such. To the contrary, about 82% of Turkish–German parents are interested in speaking Turkish within the home context (Fick et al., 2014). Previous studies have shown that – besides German – the input their children receive is indeed predominantly in Turkish (Rinker et al., 2016). However, little is known about their L1 and L2 competencies, nor about the predictors that promote DLLs’ language development in both languages, even though there is accumulating evidence that being fluent in two languages has clear advantages. Barac et al. (2014) summarize the advantages in terms of cognitive skills, particularly those skills related to inhibitory/executive control and metalinguistic awareness. Although it is known that being fully bilingual can be of great benefit, the processes and predictors that help DLLs achieve full bilingualism remain unclear. Existing research underlines the influence of some major determinants of DLLs’ L1 and L2 development, such as the quality and quantity of children’s language input at home/school, and individual or family factors (Bohman et al., 2010; Hammer et al., 2014). However, these studies have largely been conducted with older children and come from the second language acquisition or foreign language teaching traditions (Winsler et al., 2014).

In addition, recent research in Germany has primarily focused on language proficiency in L2 and findings reveal that L2 children are not proficient in their second language, which for Turkish–German children is German, the language of formal education in Germany (Schöler et al., 2006). For example, Caspar and Leyendecker’s (2011) study found lower expressive and comprehension skills in L2 (German) among Turkish–German children with Turkish as their L1. There are also some studies investigating the language skills of Turkish–German DLLs in their L1, but such studies are limited with regard to specific language levels, and in particular to (receptive) vocabulary (e.g., Willard et al., 2015). However, no study to date has comprehensively examined DLLs’ skills in their L1 within the German context. Interestingly, research in the Netherlands and the United Kingdom on the L1 skills of DLLs in those countries has also reported lower L1 proficiency (Verhoeven, 1994; Scheele et al., 2010; Haman et al., 2017). Similarly, Akoğlu and Yağmur (2016) have found that Turkish–Dutch children achieve lower L1 skills on lexical, syntactical and semantical levels.

Significantly, all of the aforementioned studies on DLLs have addressed language proficiencies and the conditions of successful language development for each language separately. Thus, as outlined in Bedore et al. (2012), there may be a potential to underestimate language skills as a result. In order to get an accurate picture of the vocabulary development of bilingual toddlers, Pearson et al. (1993) and Rinker et al. (2016) used double-language measures (Total Vocabulary and Total Conceptual Vocabulary). These measures were combined scores constructed by adding up the child’s language abilities in both languages. Total Conceptual Vocabulary counts words in both languages but denotes the same concept only once (Rinker et al., 2016). Inspired by these studies, the present study pursues a comprehensive approach with a combined language score.

### Theoretical Approaches of Dual Language Development

Although the maintenance of the learner’s L1 is a hotly debated issue, children’s first language proficiency can be an important factor for their successful L2 acquisition (Schwartz, 2014), especially in sequential bilinguals (Winsler et al., 2014). Based on Cummins (1979, 1991) “interdependence hypothesis” there is some evidence suggesting transfer effects from L1 to L2 competencies (Durgunoğlu et al., 1993; Lindsey et al., 2003; Dickinson et al., 2004; Proctor et al., 2006). The hypothesis assumes that two languages share and build upon a common base of language skills, which can be developed through high-level L1 skills and sufficient exposure to the L2. According to Cummins (1979, 1991, 2008) approach, for bilingual development to be successful, maintenance of the L1 is of great importance in avoiding the negative effects from high exposure to the L2. So, transfer mechanisms between language skills can occur via a *common underlying proficiency*. This common linguistic basis makes conceptual, metalinguistic, pragmatic and phonological knowledge accessible to both language systems (Cummins, 2008). Since common underlying proficiency underlies all languages, linguistic transfer should take place for all language combinations, largely independent of their structural similarity (language-independent transfer) (Edele and Stanat, 2016).

In contrast, the so-called time-on-task hypothesis assumes a competing relationship between the L1 and the L2 and predicts negative effects on language acquisition (Gathercole, 2002; Scheele et al., 2010). *Time-on-task* is understood as the time that a student actively spends on learning a particular thing (Hopf, 2005). It is assumed that the amount of time-on-task that is possible for L1 acquisition is not available for L2 acquisition (Edele, 2016), thereby resulting in negative consequences for L2 acquisition.

In addition to these hypotheses, the literature also discusses different linguistic models in terms of bilingual language processing. The question of how bilinguals manage their two languages, or how specific linguistic components can be selected while the others are simultaneously inhibited, remains unclear. While it has been posited that all linguistic information share a common mental lexicon, it has also been stated that there are two language nodes or language membership representations (one for each language containing information regarding the language to which an item belongs). Van Heuven et al.’s (1998) Bilingual Interactive Activation Model (BIA), the revised versions BIA + Model (Dijkstra and Van Heuven, 2002) and Multilink model (Dijkstra et al., 2018) are based on the conceptualization of two language nodes or language membership representations.

Kroll and Stewart (1994) developed another well-known model, the Revised Hierarchical Model (RHM), which assumes two separate lexicons. The L1 and L2 words and their corresponding concepts are represented in the model as linked to each other. The links between the concepts and the L1 words are the strongest. When new L2 words are learned, a link emerges between these words and the corresponding L1 words, and direct links to the concepts arise with a higher level of competence in the L2. However, this model has been criticized for being unspecific (Brysbaert and Duyck, 2010). Grainger et al. (2010) combined the RHM with the BIA model to create a developmental BIA model (BIA-d). According to this model, there are two separated lexicons during the earlier stages of L2 proficiency, as the RHM suggests. However, as L2 proficiency increases L2 lexical representations are integrated into a common lateral inhibitory network which contains words from both languages.

Regardless of whether the two lexicons are independent or integrated, there is a wide consensus among researchers (Bialystok, 2001) that both languages are active when one of them is being used (Kroll et al., 2008; Oker and Akinci, 2012). Although RHM and BIA-d models are based on a different population than the one considered in the present study (i.e., late L2 learners), they make interesting predictions about the dynamics of language development as L2 proficiency increases. However, they neither consider the role of environmental factors, such as language input at home/early daycare, nor other language domains (e.g., grammar, phonological abilities). Nevertheless, a burgeoning body of literature supports the notion that there is a relation between the two languages.

### Predictors of (First and) Second Language Acquisition

As mentioned above, there is a substantial body of evidence that DLLs’ linguistic performance is related to the qualitative and quantitative input they receive and strongly influenced by the DLLs’ own abilities. Studies imply that home language environments with large numbers of children’s books (Whitehurst et al., 1994) or activities such as shared book reading or storytelling can foster children’s language proficiency (Prevoo et al., 2014; Willard et al., 2015). However, many DLLs are children from low socio-economic status (SES) families, which offer home environments with fewer language stimulating activities (Scheele et al., 2010). SES is a strong predictor of child outcomes (Halle et al., 2012) and vocabulary development in DLLs (Mancilla-Martinez and Lesaux, 2011; Prevoo et al., 2014; Ansari and Winsler, 2016). Often families with low SES talk less to their children and tend to use a more limited range of vocabulary and grammatical structures (Hoff, 2013). In addition, L2 exposure in the context of the home is a significant positive predictor for DLLs’ L2 development (Hammer et al., 2014). Vagh et al. (2009) observed that the vocabulary size of Spanish-English bilingual toddlers who hear and use relatively more English (L2) than Spanish (L1) is closer to that of monolinguals (native speakers of English) compared to the vocabulary size of bilingual toddlers hearing and using more of their L1. Studies also point out that success in the heritage language can facilitate L2 learning (Atwill et al., 2007). According to De Houwer (2007), heritage language use in familial contexts can also be an important factor for success in both languages. A balanced use of both languages is more visible in siblings’ conversations (Caspar and Leyendecker, 2011).

Moreover, nonfamilial contexts can be useful in terms of language development, especially for migrant children who get only limited input in the L2 at home. Previous studies have identified a positive impact on the language proficiency of children in this regard (Currie, 2001; Halle et al., 2012). Espinosa (2007) also underlines the role of early education and care, since daycare facilities offer more qualitative L2 experiences compared to input usually received at home.

There is also considerable evidence that factors within the child, especially the child’s abilities, are influential in L1 and L2 language acquisition. Above all, there is a great consensus about the crucial role of phonological memory in the early years (Verhagen and Leseman, 2016). Children with stronger phonological memory skills are able to acquire a language more rapidly (Gathercole, 2006), which also applies for bilingual development (Cheung, 1996; Parra et al., 2011). There is also evidence for associations between phonological memory and grammar skills in DLLs (e.g., Verhagen and Leseman, 2016).

### The Current Study

The aim of the present study was to gain better understanding of the conditions necessary to become a functioning DLL under the assumption of an existing underlying general language proficiency using the example of Turkish–German children living in Germany. In contrast to most previous studies using one measure as a language indicator, we examined L1 and L2 language performances in different language domains (expressive and receptive vocabulary, phonological memory, and grammar) and the relations between the language competencies. Moreover, the second goal of the study was to investigate child- and language-environment-related predictors of functioning dual language development. On that account, we considered both languages together by using a form of language combination inspired by earlier investigations focused on the L1 and L2 vocabulary skills of young DLLs. Our research questions were:

1. How well developed are children’s language skills regarding their L2 (German), compared to their L1 (Turkish)? Based on previous results (Caspar and Leyendecker, 2011; Akoğlu and Yağmur, 2016), we hypothesized that bilingual children would get lower scores in their L1 and L2 compared to their monolingual peers while expecting higher levels of L1 skills than L2 skills.
2. What relation exists between the children’s L1 and L2 competencies? Based on Cummins’ transfer hypothesis (Cummins, 1979, 2008) we expected positive relations between phonological memory skills and other language domains, whereas, in accordance with the time-on-task hypothesis (Gathercole, 2002; Scheele et al., 2010), we expected negative relations between input-related language domains, such as vocabulary and grammar skills.
3. To what extent are child- and language-environment-related predictors associated with the language competencies? We expected that higher levels of phonological memory, higher SES of the family, balanced use of both languages in familial context, stimulating home literacy environments and early daycare attendance would positively predict higher language competencies.

## Materials and Methods

### Participants and Procedures

The sample (mostly consisting of sequential DLLs) involved *N* = 69 Turkish-speaking DLLs (33 girls; 36 boys; *M*_age_ = 54 months; *SD* = 10. 41 months) whose parents stated that Turkish was actively present as a family language in their home contexts. All Turkish–Kurdish children were removed from the sample if Kurdish was declared as an additionally used language. Children’s language skills were assessed in German and Turkish during a 30–45 min session. Well-trained graduate and undergraduate students and native bilingual assessors conducted the assessments on different days. Written and informed consent was obtained from the parents or legal guardians of all participants. In exchange for their participation, children received a toy for each session and parents received 10 € for filling out the parental questionnaire as a token of appreciation for their time. Among the parents who completed the questionnaire (*n* = 54; 78%), approximately half (*n* = 29; 54%) completed it in German and the remaining parents completed it in Turkish (*n* = 25; 46%). Additionally, some questionnaires had also items which have been omitted, so we have differing missing value proportions.

Information regarding country of birth was reported for 52 out of 69 (75%) parents and 47 out of 69 (68%) children. Of the parents who designated “country of birth”, 96% (*n* = 45) of the children, 37% (*n* = 19) of their mothers, and 36% (*n* = 19) of their fathers were born in Germany. The average length of residency in Germany of parents born in Turkey was 17.3 years (*SD* = 11.1) for mothers and 24.3 years (*SD* = 12) for fathers.

Only 49 out of 69 (71%) parents reported on Turkish exposure of their children. 94% (*n* = 46) of the children were exposed to Turkish, their L1, from birth, while 6% (*n* = 3) of them had first been exposed to Turkish between the ages of 12–24 months. 42 (61%) of the parents reported on German exposure. 45% (*n* = 21) of the children had contact with German from birth and 45% (*n* = 21) of children first came in contact with the German language after the age of 20 months.

Families provided information on the use of Turkish and German in the family (see Table 1). All quoted percentages and number of children (*n*) in Table 1 refer to completed items.

**Table 1:** Proportions of language use at home.

	N	Only/	Both	Only/
		mostly Turkish	languages	mostly German
Child	53	21% (*n* = 11)	78% (*n* = 41)	1% (*n* = 1)
Father	52	42% (*n* = 22)	46% (*n* = 24)	12% (*n* = 6)
Mother	52	42% (*n* = 22)	44% (*n* = 23)	14% (*n* = 7)
Siblings	43	21% (*n* = 9)	54% (*n* = 23)	25% (*n* = 11)

### Measures

Primary caregivers completed a questionnaire, available in Turkish or German, on child and family characteristics. As a measure of socioeconomic status (SES), the International Socio-Economic Index of Occupational Status (ISEI, Ganzeboom and Treiman, 1996), which is based on parents’ job titles and job descriptions, was used. Since preliminary analyses of this sample indicated that languages used by mothers and siblings are more influential to children’s language proficiency than fathers’ language use, a single dichotomous language contact variable was created. As a proxy for home literacy environment, the number of (all) available children’s books at home was recorded. The descriptive statistics are summarized in Table 2.

**Table 2:** Descriptive statistics for variables of interest.

Variable	*n*	%
**Language use of siblings and mothers**		
Balanced use of two languages	12	17
Use of one language	40	58
**Early daycare entry**		
Before the age of 30 months	13	19
After the age of 30 months	40	58
	***M***	***SD***
SES (HISEI)	40.4	12.4
Number of children’s books at home	12.1	11.2

#### Child Outcomes: Language Skills

As of now, there are no language proficiency tests that have parallel versions in German and Turkish and meet the standards of test translation and validation for the intent of comparing performances across languages. That is why language competencies in L1 and L2 were assessed via standardized language development tests designed and normed for monolingual children.

##### Receptive vocabulary

Children’s receptive vocabulary was measured using the German version of the Peabody Picture Vocabulary Test-4 (PPVT-4; Lenhard et al., 2015) and the receptive vocabulary subtest of the Turkish Expressive and Receptive Language Test (TIFALDI; Berument and Güven, 2010). The PPVT is a well-established measure of receptive language in which children choose one target image out of four. Items were presented in order of increasing difficulty and testing was stopped when the child’s response to 8 or more items within a set of 12 items was incorrect. Both measures provide raw scores and age-normed standard scores [T-score (*M* = 50, *SD* = 10) for PPVT-4 vs. Standard scores (*M* = 100, *SD* = 15) for TIFALDI, which were converted to T-scores].

##### Expressive vocabulary

Children’s expressive vocabulary was established by the AWST-R (Test for Expressive Vocabulary in German; Kiese-Himmel, 2005) and the expressive vocabulary subtest of TIFALDI (Berument and Güven, 2010). These primarily measure the expressive vocabulary skills by asking children to provide a label for pictured items. Both measures yield raw scores that can be converted to age-normed standardized scores [T-scores for the AWST-R with *M* = 50; *SD* = 10 and Standard scores *M* = 100, *SD* = 15 for TIFALDI, which were converted to T-scores].

##### Grammar

As a global grammar measure, sentence repetition tasks were used in both languages. These require children to remember sentences of increasing syntactic complexity and to use sentence meaning to assist in oral recall. The German subtest sentence repetition of HASE (Auditive Screening Battery of Heidelberg; Schöler and Brunner, 2008) provides age-standardized T-scores (*M* = 50; *SD* = 10) in addition to raw scores; however the Turkish sentence repetition subtest of TODIL (the Turkish adaptation of Test of Language Development- Primary: Fourth Edition-TOLD-P:4) (Newcomer and Hammill, 2008; Topbaş and Güven, 2017) provides raw scores and only scaled scores (*M* = 10; *SD* = 3) as age-normed standardized scores (unconvertable to T-scores).

##### Phonological memory (PM)

Nonword repetition tasks were used to assess the children’s ability to represent new phonological patterns in phonological memory. The stimuli consisted of 18 German-like nonwords (for children older than 4 years; 13 words for older than 3 years) (e.g., Billop, Defsal) and 16 Turkish-like nonwords (e.g., Tekün, Celit). The German task was taken from a German test battery for the assessment of language development in preschool children (SETK 3-5; Grimm et al., 2010). The Turkish nonword task (Topbaş et al., Unpublished) was developed in the “Cost Action IS0804: ‘Language Impairment in a Multilingual Society: Linguistic Patterns and the Road to Assessment”’ project and has as of now no norms. The children were instructed in both measures to repeat nonwords which differed in length (two to five syllables). Their performance was rated by the number of correctly recalled nonwords. To get one PM indicator, a combined score was computed by the mean of children’s *z*-standardized raw scores of nonword repetition tasks (*r =* 0.51, *p* < 0.01), since only the German task provides an age-normed score.

### Statistical Procedure

In order to explore the relation between the language measures of L1 and L2, partial correlation analyses were used. Second, to predict the DLLs’ language competencies, three separate multiple regressions were run. A composite score for language competencies as dependent variables was computed by aggregating the means of all children’s *z*-standardized expressive, receptive vocabulary and sentence repetition results (*r*s = 0.56-0.87, *p* < 0.001, among the three subtests) for each language. All measures of L1 and L2 were used for the dual language competence score. For the regression analyses, information on age, phonological memory, SES, balanced use of languages by mothers and siblings, number of available children’s books at home and early entry to a daycare facility were used as independent variables. These independent variables were selected since previous analyses showed significant correlations with at least one of the aggregated language scores.

Missing data (ranging from 1.4 to 34.8%) was due to response omissions, parents not filling in parts of questionnaires, and the frequent absence of children. Missing value analysis and Little’s Missing Completely at Random (MCAR) test suggested that the data were consistent with the pattern of MCAR (χ^2^ = 11.54, *p* > 0.05). In terms of using all available data for estimating the parameters and increasing the statistical power, missing data were dealt by multiple imputations (*m = 5*; Little and Rubin, 2002; Carpenter et al., 2013; Lüdtke et al., 2017). Multiple imputation is a recommended strategy for addressing missing data problems in small sample sizes with high missing data proportions of up to 50% (Graham and Schafer, 1999). All statistical analyses and multiple imputations were conducted in SPSS Version 22 (IBM Corp, 2013).

## Results

### Language Proficiencies in First and Second Language

Descriptive data for the measures of language skills in Turkish (L1) and German (L2) are presented in Table 3. Since not every test used age-normed standardized scores, raw scores from the tests were used in analyses for the second and third questions. Table 3 contains also the age-normed scores. However, it should be noted that all of the measures used were normed and developed for monolingual children.

**Table 3:** Descriptive language proficiencies.

Variable	N	L1			L2	
				
		Raw score	T-score/Scaled score for grammar	N	Raw score	T-score
		Mean (*SD*)	Mean (*SD*)		Mean (*SD*)	Mean (*SD*)
Receptive vocabulary	69	36.48 (18.58)	49.45 (7.56)	66	42.18 (28.43)	32.55 (6.66)
Expressive vocabulary	59	26.25 (15.00)	46.73 (10.07)	67	9.66 (9.66)	24.93^a^ (6.91)
Grammar	63	2.55 (2.80)	5.58^b^ (1.67)	62	2.40 (1.91)	29.60^a^ (7.77)
Phonological memory	64	7.35 (3.33)	–^c^	62	7.35 (3.95)	44.16 (9.67)

*^a^Since publishers provide only norms for specific age groups, these results include the subgroup of children aged between 3;00 and 5;05 years for German expressive vocabulary (AWST-R). German grammar T-scores (HASE) were also reported for children aged only between 4;06 and 6;11 years.*

*^b^TODIL provides only scaled scores as age-normed scores, which are not convertible to T-scores.*

*^c^Turkish nonword repetition tasks have no norms.*

With the exception of phonological memory, the sample demonstrates lower levels of language proficiency in German (L2). The mean T-scores for vocabulary and grammar scores in German were below the lower limits of the normal range. Children’s German nonword repetition scores were higher than other results and within average range (*T* = 44.16; *SD* = 9.67). By contrast, participants had higher mean scores in Turkish in expressive and receptive vocabulary in particular. Based on T-scores, children showed age appropriate vocabulary levels in Turkish and they were significantly better than German vocabulary skills [Receptive Vocabulary: *t*(65) = 13.39, *p* < 0.001; Expressive Vocabulary: *t*(41) = 10.06, *p* < 0.001]. Since no norms exist for the Turkish nonword repetition task, a comparison with monolingual Turkish children was not possible. The scaled score of Turkish sentence repetition results shows that the mean performance level in Turkish grammar was below the norming sample mean (*M* = 10; *SD* = 2), ranging from 3 to 10 scaled score points.

### Relationships Between First and Second Language Measures

To examine the second question, concerning the relationships between the two languages, bivariate partial correlations with age as covariable were explored. As Table 4 demonstrates, expressive vocabulary in L1 showed significant negative correlations with most measures of L2: the higher expressive vocabulary is in one language, the lower it becomes in the other language. Additionally, phonological memory results were moderately correlated across most language skills in L1 and L2. There were no more significant cross-language correlations present.

**Table 4:** Bivariate (partial) correlations of L1 and L2 proficiencies (controlled for age).

	Receptive vocabulary (L2)	Expressive vocabulary (L2)	Grammar (L2)	Phonological memory (L2)
Receptive vocabulary (L1)	-0.10	-0.10	-0.10	0.28^∗^
Expressive vocabulary (L1)	-0.40^∗^	-0.41^∗^	-0.41^∗^	0.09
Grammar (L1)	0.04	-0.08	-0.04	0.34^∗^
Phonological memory (L1)	0.26^∗^	0.27^∗^	0.27^†^	0.51^∗∗^

*^†^p < 0.10; ^∗^p < 0.05; ^∗∗^p < 0.01.*

### Prediction of Language Competencies

To pursue our third research question, three separate multiple regression analyses were run using children’s age and phonological memory, SES, variables of siblings’ and mothers’ balanced use of both languages, the number of children’s books at home and early daycare entry as predictors. Results (presented in Table 5) revealed that six variables included in Model 1 explained 75% [adj. *R*^2^ = 0.75, *F*(6, 62), *p* < 0.001] of variance in the children’s dual language proficiency. Age, PM, and early entry to daycare facilities were significant predictors of language skills in both languages. Children who demonstrated higher levels of PM had higher dual language proficiency. Early daycare attendance was also a significant positive predictor, showing that Turkish–German DLLs who enter daycare facilities before the age of 30 months had better dual language abilities. SES, siblings’ and mothers’ balanced use of both languages in their home contexts and the number of children’s books at home were not significant predictors.

**Table 5:** Multiple regression predicting Turkish–German DLL’s dual, German and Turkish Proficiency in Kindergarten.

Predictors	Dual language competence (Model 1)		German competence (Model 2)		Turkish competence (Model 3)	
	*F*(6, 62) = 32.99^∗∗∗^		*F*(6, 62) = 17.02^∗∗∗^		*F*(6, 62) = 8.43^∗∗∗^	
	Adj. *R*^2^= 0.75		Adj. *R*^2^ = 0.60		Adj. *R*^2^ = 0.42	
	β	*p*	β	*p*	β	*p*
Age	0.57^∗∗^	<0.01	0.27^∗^	0.02	0.31^∗^	<0.05
Phonological memory	0.78^∗∗∗^	0.00	0.42^∗∗^	0.00	0.36^∗^	0.03
SES	-0.04	0.78	-0.01	0.91	-0.03	0.88
Siblings’ and mothers’ balanced used of both languages	0.08	0.49	0.10	0.25	-0.02	0.87
Number of children’s books at home	0.11	0.45	0.31^∗∗^	0.01	-0.20	0.10
Daycare entry before 30 months	0.28^∗^	0.03	0.22^∗^	0.03	0.06	0.59

*Standardized b coefficients and p-values were derived from pooled estimates of parameters (weighted by standard errors) from 5 imputed data sets. R^2^ statistics and analysis of variance were estimated from an aggregated data consisting of the mean of imputed data. Continuous predictors and covariates were centered at their means. Variables of siblings’ and mothers’ balanced use of both languages and early daycare entry were dummy coded (Yes = 1, No = 0).*

*^∗^p < 0.05; ^∗∗^p < 0.01; ^∗∗∗^p < 0.001.*

Six variables included in the model to explain L2 competencies explained 60% of the variance in German language competence [adj. *R*^2^ = 0.60, *F*(6, 62), *p* < 0.001]. Children with higher PM abilities, early daycare entry, and higher numbers of available children’s books at home scored higher in German language tests. Neither SES nor siblings’ and mothers’ balanced use of both languages in their home contexts was a significant predictor.

Only age and phonological memory had a significant effect on Turkish language competencies [adj. *R*^2^ = 0.42, *F*(6, 62), *p* < 0.001]. Other entered variables were unable to explain any significant additional variance in Turkish competencies.

In order to evaluate the possible effect of predictors, which did not become significant within the final regression model, *t*-Tests were conducted to examine mean differences in language competencies. Analyses indicated that children with balanced language use at home (*M* = 0.66; *SD* = 1.00) showed significantly higher German language competencies [*t*(49) = 2.25, *p* = 0.03] than children with unbalanced language use (*M* = -0.07; *SD* = 0.94) at home. In contrast, no significant differences in terms of Turkish competencies [*M* = 0.02; *SD* = 1.08 vs. *M* = -0.01; *SD* = 0.81; *t*(46) = -0.01, *p* = n.s.] and dual language competencies [*M* = 0.02; *SD* = 1.39 vs. *M* = 0.80; *SD* = 1.58; *t*(45) = -1.58, *p* = n.s.] were found when both languages were used in the home context.

## Discussion

This is the first study in Germany which offers a detailed insight to the language skills of Turkish–German children. It marks the first exploration of the L1 and L2 performance of children aged 3 to 6 in different language domains and the interrelations between said domains. In addition, the study investigated how functioning dual language development can be achieved by trying to predict the language competencies of Turkish–German DLLs based on selected family and child factors – an understudied topic in our field.

As hypothesized, lower L2 skills and lower L1 grammar skills (sentence repetition) were identified. These results are in line with earlier research observing lower language levels in L1 and L2 (e.g., Caspar and Leyendecker, 2011; Akoğlu and Yağmur, 2016). However, our sample consisted of children with well-developed vocabulary skills in their L1, even when compared with monolingual norms. We assume that this is a result of the (often reported) preference of the Turkish community to maintain their heritage language (Extra and Yagmur, 2010; Eversteijn-Kluijtmans, 2011). It is known that young Turkish DLLs are often primarily exposed to Turkish in their very early childhood until they enter a childcare institution or school (Becker, 2010), a situation evident in our sample. “Access to Turkish media, holidays in Turkey, life partners from Turkey (and) high numbers of Turkish organizations” as described by Bezcioglu-Goktolga and Yagmur (2017, p. 47), play an important role in terms of maintaining the L1 among the Turkish community (Backus, 2013).

Our findings only partially support the assumption that well-developed L1 competencies indicate better L2 competencies. With the exception of phonological memory and expressive vocabulary of L1, children’s L1 and L2 skills had no relationships with each other. These findings align with studies reporting inconsistent cross-linguistic effects (e.g., Verhoeven, 1994; Scheele et al., 2010). The results concerning expressive vocabulary indicate a trend toward a competition between these language measures, which are well known to be strongly input-related. Thus, this result appears to be in line with the time-on-task hypothesis (Gathercole, 2002; Scheele et al., 2010), which suggests a competitive relation between the learning time and proficiency for L1 and L2. Further analyses are necessary in order to take into account the aspects of the children’s language use and that of their families, and thus to get a clearer picture. The present results offer mere correlations without considering other related aspects: for this reason, no firm conclusions can be drawn.

On the contrary, significant positive correlations between phonological memory skills and all other language domains seem to support our assumption of common underlying language ability (Cummins, 2008). We assume that these abilities tend to be independent of any particular knowledge and can be seen as at least a component of that common underlying cognitive capacity (Parra et al., 2011). Moreover, our study reveals that cross-linguistic transfer is not limited to languages with high similarity, but also applies to more distant languages such as German and Turkish (Edele and Stanat, 2016). These results also support our hypothesis that phonological memory would be predictive for L1, L2 and dual language proficiency, a hypothesis based on several prior studies outlining similar associations (Cheung, 1996; Kormos and Sáfár, 2008; Parra et al., 2011; Verhagen and Leseman, 2016). However, it should be noted that these studies mostly addressed specific aspects of language proficiency (e.g., vocabulary and grammar). There are also studies inferring positive associations between phonological memory and vocabulary, but no predictive relations between phonological memory and grammar abilities (Engel de Abreu and Gathercole, 2012). Consequently, it is possible to trace our findings back to the relations between phonological memory and vocabulary.

No association between SES and language proficiency could be found in our study for Turkish or German, but an association between SES and L1 and L2 skills has been reported in previous studies (Halle et al., 2012; Hoff, 2013). This is probably because our sample mostly consisted of families with lower SES level. In addition, it is well known that the quality and amount of input are more important than parents’ job status or income level. Prevoo et al. (2014) and Arriagada (2005) have suggested that SES has an influence on language use and shapes home language context of families, but further research is needed to identify the mechanisms influencing the associations between SES and language competencies.

Current research concludes that there is no evidence that a strict one-person-one language principle is essential for successful bilingual development (De Houwer, 2007; Hoff and Core, 2013) and no evidence for detrimental effects of a lack of separation of two languages in the longer term (Hoff et al., 2014). Our results reveal no adverse effects of balanced language use in bilingual homes (meaning mixed language use/code-switching within the family) for all language competencies and for L1 skills in particular. Moreover, both initial correlation analyses and *t*-Test analyses support the assumption that this predictor shows a positive association with at least German language competencies (and no adverse effects on Turkish competencies) although it was no longer significant after adding other predictors to the complex regression models. A study of 18 month olds suggests a negative relation between parents using words from both languages in one sentence and their receptive vocabulary (Byers-Heinlein, 2013). Accordingly, code-switching within individual sentences does not necessarily seem to be conducive to strengthening the language skills of DLLs. On the other hand, it is probably not essential to separate the languages or to avoid one of the languages in order to achieve competent dual language development (Hoff and Core, 2013).

Different studies have proposed that home language activities such as book reading predict children’s cognitive and language skills (Raikes et al., 2006; Mol et al., 2008). The availability of children’s books at home, an often-used proxy for home language activities, was used to predict the children’s language competencies. Results indicate that the number of children’s books was related only to the children’s German language competencies. A possible explanation for these results is the fact that most books were in German. This may be a result of challenges accessing books and other reading materials in Turkish, which makes it difficult for families to include book-reading activities in parent–child interactions. Furthermore, it is known that dyadic book reading is a culture-specific activity (Perry et al., 2008) and not common practice among Turkish immigrant families (Jäkel et al., 2011).

Early daycare attendance has an effect only on the children’s dual language and German proficiency. The prediction for dual language proficiency can be attributed to the fact that early daycare attendance is of great importance for the development of L2 competencies. Nevertheless, the association between early daycare entry and dual language proficiency is higher than for the prediction of only German language competencies and it has no detrimental effects on the L1 Turkish. Previous studies indicate that early daycare attendance is a strong predictor of high-level language competencies (Becker, 2010; Yazejian et al., 2015). Multi/bilingual day-care facilities that attempt to involve children’s home languages in the classrooms are also increasingly recommended. The advantages of such approaches have been proposed not only for L2 acquisition with no negative impact on the heritage language (Buysse et al., 2014), but also for the socio-emotional development of DLLs (Halle et al., 2014). It is essential to note that children who entered daycare facilities before the age of 30 months also had higher familial SES. This leads to the assumption that early daycare attendance was a result of family constellations in which both parents were working. Our findings also support the fact that children with Turkish migration background often enter preschools later than German children. A study in Germany found out that one of the reasons for the underrepresentation of migrant children in early daycare facilities is that the majority of migrant mothers want to bring up their children themselves (Sachverständigenrat deutscher Stiftungen für Integration und Migration, 2013). Turkish mothers may prefer homecare, staying at home and giving up their employment status, over working in a low-status job.

## Conclusion

Taken together, our results indicate that DLL’s competencies in each language do not develop independently of one another. We were able to show positive relations for phonological memory skills, and to demonstrate negative relations for expressive vocabulary skills in L1. These results underline the importance of taking a closer look at their relations while also taking other factors into account. They also demonstrate that mothers’ and siblings’ balanced use of both languages and early daycare entry do not cause any detrimental effects on children’s language skills. Functioning dual language development depends on opportunities to use and hear both languages. With respect to successful bilingual development, it is important to take both languages into consideration, not only for children’s general wellbeing but also in order to make statements about DLLs’ language development. One of the strengths of our study is that we examined language proficiency in both languages and in different language domains for the purpose of capturing as complete a picture as possible. Although our research is insightful, more research is needed in order to fully understand the factors contributing to functioning dual language development and to operationalize the concept of dual language competencies. Additionally, the construct *dual language competencies* should also be critically scrutinized, since our approach enables a child with above-average competencies in only one language to achieve the same value as a child with average levels in both languages. A study with a larger sample would lead to a more precise empirical basis for defining what functioning bi- or multilingual development means and implies. Such fundamental research knowledge can serve as a foundation for programs to support multilingual families as well as for practical work with DLLs.

### Limitation

This study’s findings must be considered in light of its limitations. First, our study had a correlational and cross-sectional design. Examining the relations and dual language development in a longitudinal way would shed further light on questions about how dual language development can be achieved and would help to draw firm conclusions about the causality of the effects. Moreover, it would also inform our understanding of the extent to which the often-reported slow patterns of development in L1 and L2 are rooted in child- and language-environment-related conditions. Second, the language proficiency tests we used were not designed for assessing language skills of bilingual children, nor do all of them provide norms for monolingual and bilingual children. But high to moderately high correlations between the measures indicate that this approach should not be a major limitation. Our study strongly supports the notion that specific language tests or at least bilingual norms are required in order to avoid underestimating children’s skills or mistakenly classifying those with normal development as at-risk children. In addition, it is also important to point out that the nonword repetition tasks used measure more than memory abilities; high correlations between the phonological memory variable and the dependent variables of the regression analyses indicate that language abilities cannot be separated from that construct. That is why in further studies an additional task – such as a more language-independent digit recall task (or currently discussed language-independent nonwords) – could be used in order to control confounding effects. The third limitation of this study concerns the proportion of missing data. Although we have used a highly recommended method for dealing with missing values (Lüdtke et al., 2017), the best way to handle this is to do everything possible to avoid missing observations in the first place. Face-to-face interviews with the principle caregivers may help to prevent much missing information and permit a greater level of detail regarding the child and their family environment as well.

## Ethics Statement

This study was carried out in accordance with the recommendations of the German Research Foundation’s Commission “Self-Regulation in Science for safeguarding good scientific practice”, of the German Rector’s Conference, based on resolutions from the senate of the Max-Planck-Society published under the title “Rules of Procedure in Cases of Suspected Scientific Misconduct”, and the “Guidelines for the safeguarding of good scientific practice” of the Heidelberg University by ethics committee of Heidelberg University of Education. The protocol was approved by the ethics committee of Heidelberg University of Education. All subjects gave written informed consent in accordance with the Declaration of Helsinki.

## Author Contributions

JK and SS: leadership and design of the study. SS and BE: design of tasks used in the study, data analysis, and data interpretation. BE, JK, MF, SJ, and SS: data collection. BE: manuscript authorship. JK and SS: critical revision and editing.

## Conflict of Interest Statement

The authors declare that the research was conducted in the absence of any commercial or financial relationships that could be construed as a potential conflict of interest.

## References

[B1] Akoğlu G. Yağmur K. (2016). First-language skills of bilingual Turkish immigrant children growing up in a Dutch submersion context. *Int. J. Biling. Educ. Biling.* 19 706–721. 10.1080/13670050.2016.1181605

[B2] Ansari A. Winsler A. (2016). Kindergarten readiness for low-income and ethnically diverse children attending publicly funded preschool programs in Miami. *Early Child Res. Q.* 37 69–80. 10.1016/j.ecresq.2016.06.002 PMC9161748 35662914

[B3] Arriagada P. A. (2005). Family Context and Spanish-Language Use. A Study of Latino Children in the United States. *Soc. Sci. Q.* 86 599–619. 10.1111/j.0038-4941.2005.00320.x

[B4] Atwill K. Blanchard J. Gorin J. S. Burstein K. (2007). Receptive vocabulary and cross-language transfer of phonemic awareness in kindergarten children. *J. Educ. Res.* 100 336–346. 10.3200/JOER.100.6.336-346

[B5] Autorengruppe Bildungsberichterstattung (2016). *Bildung in Deutschland 2016. Ein indikatorgestützter Bericht mit einer Analyse zu Bildung und Migration [Education in Germany 2016. An Indicator-based Report with an Analysis on Education and Migration].* Bielefeld: Bertelsmann.

[B6] Backus A. (2013). “Turkish as an immigrant language in Europe,” in *The Handbook of Bilingualism*, eds Bhatia T. K. Ritchie W. C. (Malden, MA: Blackwell Publishing), 770–790.

[B7] Barac R. Bialystok E. Castro D. C. Sanchez M. (2014). The cognitive development of young dual language learners: a critical review. *Early Child Res. Q.* 29 699–714. 10.1016/j.ecresq.2014.02.003 25284958 PMC4180217

[B8] Becker B. (2010). Wer profitiert mehr vom Kindergarten? *Köln. Z. Soziol.* 62 139–163. 10.1007/s11577-010-0090-5

[B9] Bedore L. M. Pena E. D. Summers C. L. Boerger K. M. Resendiz M. D. Greene K. (2012). The measure matters: language dominance profiles across measures in Spanish-English bilingual children. *Bilingualism* 15 616–629. 10.1017/S1366728912000090 23565049 PMC3615884

[B10] Berument S. K. Güven A. G. (2010). *TİFALDİ Türkçe İfade Edici ve Alıcı Dil Testi.* Ankara: Kültür ve Turizm Bakanlıǧı.

[B11] Bezcioglu-Goktolga I. Yagmur K. (2017). Home language policy of second-generation Turkish families in the Netherlands. *J. Multiling. Multicult. Dev.* 2 1–16. 10.1080/01434632.2017.1310216

[B12] Bialystok E. (2001). *Bilingualism in Development: Language, Literacy, and Cognition.* Cambridge, MA: Cambridge University Press 10.1017/CBO9780511605963

[B13] Bohman T. M. Bedore L. M. Peña E. D. Mendez-Perez A. Gillam R. B. (2010). What you hear and what you say: language performance in Spanish-English bilinguals. *Int. J. Biling. Educ. Biling.* 13 325–344. 10.1080/13670050903342019 21731899 PMC3128885

[B14] Brysbaert M. Duyck W. (2010). Is it time to leave behind the Revised Hierarchical Model of bilingual language processing after fifteen years of service? *Biling. Lang. Cogn.* 13 359–371. 10.1017/S1366728909990344

[B15] Buysse V. Peisner-Feinberg E. Páez M. Hammer C. S. Knowles M. (2014). Effects of early education programs and practices on the development and learning of dual language learners. A review of the literature. *Early Child Res. Q.* 29 765–785. 10.1016/j.ecresq.2013.08.004

[B16] Byers-Heinlein K. (2013). Parental language mixing. Its measurement and the relation of mixed input to young bilingual children’s vocabulary size. *Bilingualism* 16 32–48. 10.1017/S1366728912000120

[B17] Carpenter J. R. Roger J. H. Kenward M. G. (2013). Analysis of longitudinal trials with protocol deviation: a framework for relevant, accessible assumptions, and inference via multiple imputation. *J. Biopharm. Stat.* 23 1352–1371. 10.1080/10543406.2013.834911 24138436

[B18] Caspar U. Leyendecker B. (2011). Deutsch als Zweitsprache. *Z. Entwickl. Padagogis.* 43 118–132. 10.1026/0049-8637/a000046

[B19] Cheung H. (1996). Non-word span as a unique predictor of second-language vocabulary language. *Dev. Psychol.* 32 867–873. 10.1037/0012-1649.32.5.867

[B20] Cummins J. (1979). Linguistic interdependence and the educational development of bilingual children. *Rev. Educ. Res.* 49 222–251. 10.3102/00346543049002222

[B21] Cummins J. (1991). “Interdependence of first- and second-language proficiency in bilingual children,” in *Language Processing in Bilingual Children*, ed. Bialystok E. (Cambridge, MA: Cambridge University Press), 70–89.

[B22] Cummins J. (2008). “Teaching for transfer: Challenging the two solitudes assumption in bilingual education,” in *Encyclopedia of Language and Education*, ed. Hornberger N. H. (Boston, MA: Springer), 1528–1538.

[B23] Currie J. (2001). Early childhood education programs. *J. Econ. Perspect.* 15 213–238. 10.1257/jep.15.2.213

[B24] De Houwer A. (2007). Parental language input patterns and children’s bilingual use. *Appl. Psycholinguist.* 28 411–424. 10.1017/S0142716407070221

[B25] Edele A. (2016). *Die Rolle herkunftssprachlicher Kompetenz und kultureller Identität für den Bildungserfolg von Heranwachsenden aus zugewanderten Familien. [The role of heritage language competencies and cultural identity for the educational success of adolescents from immigrant families].* Doctoral dissertation, Freie Universität Berlin, Berlin.

[B26] Dickinson D. K. McCabe A. Clark-Chiarelli N. Wolf A. (2004). Cross-language transfer of phonological awareness in low-income Spanish and English bilingual preschool children. *Appl. Psycholinguist.* 25 323–347. 10.1017/S0142716404001158

[B27] Dijkstra T. Van Heuven W. J. (2002). The architecture of the bilingual word recognition system: from identification to decision. *Biling. Lang. Cogn.* 5 175–197. 10.1017/S1366728902003012 15905078

[B28] Dijkstra T. Wahl A. Buytenhuijs F. van Halem N. Al-jibouri Z. de Korte M. (2018). Modeling bilingual lexical processing: a research agenda and desiderabilia. *Biling. Lang. Cogn.* 1–11. 10.1017/S1366728918000986

[B29] Durgunoğlu A. Y. Nagy W. E. Hancin-Bhatt B. J. (1993). Cross-language transfer of phonological awareness. *J. Educ. Psychol.* 85 453–465. 10.1037/0022-0663.85.3.453

[B30] Edele A. Stanat P. (2016). The role of first-language listening comprehension in second-language reading comprehension. *J. Educ. Psychol.* 108 84–110. 10.1037/edu0000060 2622299

[B31] Engel de Abreu P. M. J. Gathercole S. E. (2012). Executive and phonological processes in second-language acquisition. *J. Educ. Psychol.* 104 974–986. 10.1037/a0028390

[B32] Espinosa L. M. (2007). “English-language learners as they enter school,” in *School Readiness and the Transition to Kindergarten in the Era of Accountability*, eds Pianta R. C. Cox M. J. Snow K. L. (Baltimore, MD: Paul H. Brookes Publishing Co.), 175–196.

[B33] Eversteijn-Kluijtmans N. I. M. (2011). *“All at Once”: Language Choice and Codeswitching by Turkish-Dutch Teenagers.* Doctoral dissertation, Tilburg University, Tilburg.

[B34] Extra G. Yagmur K. (2010). Language proficiency and socio-cultural orientation of Turkish and Moroccan youngsters in the Netherlands. *Lang. Educ.* 24 117–132. 10.1080/09500780903096561

[B35] Fick P. Wöhler T. Diehl C. Hinz T. (2014). *Integration Gelungen? Die Fünf Größten Zuwanderungsgruppen in Baden-Württemberg im Generationenvergleich. [Successful Integration? The Five Largest Migrant Groups in Baden-Württemberg in an Intergenerational Comparison].* Konstanz: University of Konstanz.

[B36] Ganzeboom H. B. Treiman D. J. (1996). Internationally comparable measures of occupational status for the 1988 international standard classification of occupations. *Soc. Sci. Res.* 25 201–239. 10.1006/ssre.1996.0010

[B37] Gathercole S. E. (2006). Nonword repetition and word learning. The nature of the relationship. *Appl. Psycholinguist.* 27 513–543. 10.1017/S0142716406060383 15257665

[B38] Gathercole V. C. M. (2002). “Monolingual and bilingual acquisition: learning different treatments of that-trace phenomena in English and Spanish,” in *Language and Literacy in Bilingual Children*, eds Oller D. K. Eilers R. E. (Clevedon: Multilingual Matters), 220–252.

[B39] Graham J. W. Schafer J. L. (1999). “On the performance of multiple imputation for multivariate data with small sample size,” in *Statistical Strategies for Small Sample Research*, ed. Hoyle R. (Thousand Oaks, CA: Sage), 1–27.

[B40] Grainger J. Midgley K. Holcomb P. J. (2010). “Re-thinking the bilingual interactive-activation model from a developmental perspective (BIA-d),” in *Language Acquisition Across Linguistic and Cognitive Systems*, eds Kail M. Hickmann M. (New York, NY: John Benjamins), 267–283. 10.1075/lald.52.18gra

[B41] Grimm H. Aktas M. Frevert S. (2010). *SETK 3-5: Sprachentwicklungstest für drei- bis fünfjährige Kinder*, 2nd Edn. Göttingen: Hogrefe.

[B42] Halle T. Hair E. Wandner L. McNamara M. Chien N. (2012). Predictors and outcomes of early vs. later English language proficiency among English language learners. *Early Child Res. Q.* 27 1–20. 10.1016/j.ecresq.2011.07.004 22389551 PMC3290413

[B43] Halle T. G. Whittaker J. V. Zepeda M. Rothenberg L. Anderson R. Daneri P. (2014). The social–emotional development of dual language learners. Looking back at existing research and moving forward with purpose. *Early Child Res. Q.* 29 734–749. 10.1016/j.ecresq.2013.12.002

[B44] Haman E. Wodniecka Z. Marecka M. Szewczyk J. Białecka-Pikul M. Otwinowska A. (2017). How does L1 and L2 exposure impact L1 performance in bilingual children? Evidence from Polish-English migrants to the United Kingdom. *Front. Psychol.* 8:1444. 10.3389/fpsyg.2017.01444 28928681 PMC5591580

[B45] Hammer C. S. Hoff E. Uchikoshi Y. Gillanders C. Castro D. Sandilos L. E. (2014). The language and literacy development of young dual language learners: a critical review. *Early Child Res. Q.* 29 715–733. 10.1016/j.ecresq.2014.05.008 25878395 PMC4394382

[B46] Hoff E. (2013). Interpreting the early language trajectories of children from low-SES and language minority homes: implications for closing achievement gaps. *Dev. Psychol.* 49 4–14. 10.1037/a0027238 22329382 PMC4061698

[B47] Hoff E. Core C. (2013). Input and language development in bilingually developing children. *Semin. Speech Lang.* 34 215–226. 10.1055/s-0033-1353448 24297614 PMC4457512

[B48] Hoff E. Welsh S. Place S. Ribot K. (2014). “Properties of dual language input that shape bilingual development and properties of environments that shape dual language input,” in *Input and Experience in Bilingual Development*, eds Grüter T. Paradis J. (Amsterdam: John Benjamins Publishing),119–140.

[B49] Hopf D. (2005). Zweisprachigkeit und Schulleistung bei Migrantenkindern. *Z. Padagogik* 51 236–251.

[B50] IBM Corp. (2013). *IBM SPSS Statistics.* Armonk, NY: IBM Corp.

[B51] Jäkel J. Schölmerich A. Kassis W. Leyendecker B. (2011). Mothers’ and Fathers’ book reading predicts preschoolers´ development in Turkish immigrant and German families. *Int. J. Dev. Sci.* 5 27–39. 10.1111/scs.12140 24735278

[B52] Kiese-Himmel C. (2005). *AWST-R. Aktiver Wortschatztest für 3- bis 5-jährige Kinder. Manual.* Göttingen: Beltz Test.

[B53] Kormos J. Sáfár A. (2008). Phonological short-term memory, working memory and foreign language performance in intensive language learning. *Biling. Lang. Cogn.* 11 261–271. 10.1017/S1366728908003416

[B54] Kroll J. F. Bobb S. C. Misra M. Guo T. (2008). Language selection in bilingual speech: evidence for inhibitory processes. *Acta Psychol.* 128 416–430. 10.1016/j.actpsy.2008.02.001 18358449 PMC2585366

[B55] Kroll J. F. Stewart E. (1994). Category interference in translation and picture naming: evidence for asymmetric connection between bilingual memory representations. *J. Mem. Lang.* 33 149–174. 10.1006/jmla.1994.1008

[B56] Lenhard A. Lenhard W. Segerer R. Suggare S. (2015). *Peabody Picture Vocabulary Test - Revision IV (German Adaption).* Frankfurt: Pearson Assessment.

[B57] Lindsey K. A. Manis F. R. Bailey C. E. (2003). Prediction of first-grade reading in Spanish-speaking English-language learners. *J. Educ. Psychol.* 95 482–494. 10.1037/0022-0663.95.3.482

[B58] Little R. J. Rubin D. B. (eds). (2002). “Bayes and multiple imputation,” in *Statistical Analysis with Missing Data* (Hoboken, NJ: John Wiley & Sons, Inc.), 200–220.

[B59] Lüdtke O. Robitzsch A. Grund S. (2017). Multiple imputation of missing data in multilevel designs: A comparison of different strategies. *Psychol. Methods* 22 141–165. 10.1037/met0000096 27607544

[B60] Mancilla-Martinez J. Lesaux N. K. (2011). The gap between Spanish speakers’ word reading and word knowledge: a longitudinal study. *Child Dev.* 82 1544–1560. 10.1111/j.1467-8624.2011.01633.x 21848955 PMC3169767

[B61] Mol S. E. Bus A. G. de Jong M. T. Smeets D. J. H. (2008). Added value of dialogic parent–child book readings. A Meta-Analysis. *Early Educ. Dev.* 19 7–26. 10.1080/10409280701838603

[B62] Newcomer P. Hammill D. (2008). *The Test of Language Development: Primary-4.* Austin, TX: Pro-Ed.

[B63] Oker A. Akinci M.-A. (2012). Processus implicite de dénomination de mots chez des enfants bilingues franco-turcs de 5 ans. *CogniTextes.* 8 1–12. 10.4000/cognitextes.524

[B64] Parra M. Hoff E. Core C. (2011). Relations among language exposure, phonological memory, and language development in Spanish-English bilingually developing 2-year-olds. *J. Exp. Child Psychol.* 108 113–125. 10.1016/j.jecp.2010.07.011 20828710 PMC4073230

[B65] Pearson B. Z. Fernández S. C. Oller D. K. (1993). Lexical development in bilingual infants and toddlers. Comparison to monolingual norms. *Lang. Lear.* 43 93–120. 10.1111/j.1467-1770.1993.tb00174.x

[B66] Perry N. J. Kay S. M. Brown A. (2008). Continuity and change in home literacy practices of Hispanic families with preschool children. *Early Child Dev.* 178 99–113. 10.1080/03004430701482191

[B67] Prevoo M. J. L. Malda M. Mesman J. Emmen R. A. G. Yeniad N. van Ijzendoorn M. H. (2014). Predicting ethnic minority children’s vocabulary from socioeconomic status, maternal language and home reading input: different pathways for host and ethnic language. *J. Child Lang.* 41 963–984. 10.1017/S0305000913000299 24067295

[B68] Proctor C. P. August D. Carlo M. S. Snow C. (2006). The intriguing role of Spanish language vocabulary knowledge in predicting English reading comprehension. *J. Educ. Psychol.* 98 159–169. 10.1037/0022-0663.98.1.159

[B69] Raikes H. Pan B. A. Luze G. Tamis-LeMonda C. S. Brooks-Gunn J. Constantine J. (2006). Mother-child bookreading in low-income families: correlates and outcomes during the first three years of life. *Child Dev.* 77 924–953. 10.1111/j.1467-8624.2006.00911.x 16942498

[B70] Rinker T. Budde-Spengler N. Sachse S. (2016). The relationship between first language (L1) and second language (L2) lexical development in young Turkish-German children. *Int. J. Biling. Educ. Biling.* 20 218–233. 10.1080/13670050.2016.1179260

[B71] Sachverständigenrat deutscher Stiftungen für Integration und Migration (2013). *Hürdenlauf zur Kita: Warum Eltern mit Migrationshintegrund ihr Kind seltener in die frühkindliche Tagesbetreuung schicken [Hurdles to the day-care center: why parents with migrant backgrounds rarely send their children to early childhood institutions].* Berlin: Policy Brief.

[B72] Scheele A. F. Leseman P. P. M. Mayo A. Y. (2010). The home language environment of monolingual and bilingual children and their language proficiency. *Appl. Psycholinguist.* 31:117 10.1017/S0142716409990191

[B73] Schöler H. Brunner M. (2008). *Heidelberger Auditives Screening in der Einschulungsuntersuchung*, 2nd Edn. Binswagen: WESTRA.

[B74] Schöler H. Guggenmos J. Iseke A. (2006). Werden die Sprachleistungen unserer Kinder immer schwächer? Beobachtungen an sechs Einschulungsjahrgängen in Münster. *Gesundheitswesen* 68 337–346. 10.1055/s-2006-926892 16826466

[B75] Schwartz M. (2014). The impact of the First Language First model on vocabulary development among preschool bilingual children. *Read. Writ.* 27 709–732. 10.1007/s11145-013-9463-2

[B76] Topbaş S. Güven O. S. (2017). *Türkçe Okulcağı Dil Gelişimi Testi-TODIL.Test Bataryası.* Ankara: Detay Yayıncılık.

[B77] Vagh S. B. Pan B. A. Mancilla-Martinez J. (2009). Measuring growth in bilingual and monolingual children’s English productive vocabulary development: the utility of combining parent and teacher report. *Child Dev.* 80 1545–1563. 10.1111/j.1467-8624.2009.01350.x 19765017

[B78] van Heuven W. J. Dijkstra T. Grainger J. (1998). Orthographic neighborhood effects in bilingual word recognition. *J. Mem. Lang.* 39 458–483. 10.1006/jmla.1998.2584

[B79] Verhagen J. Leseman P. (2016). How do verbal short-term memory and working memory relate to the acquisition of vocabulary and grammar? A comparison between first and second language learners. *J. Exp. Child Psychol.* 141 65–82. 10.1016/j.jecp.2015.06.015 26340756

[B80] Verhoeven L. T. (1994). Transfer in bilingual development. The linguistic interdependence hypothesis revisited. *Lang. Lear.* 44 381–415. 10.1111/j.1467-1770.1994.tb01112.x

[B81] Whitehurst G. J. Arnold D. S. Epstein J. N. Angell A. L. Smith M. Fischel J. E. (1994). A picture book reading intervention in day care and home for children from low-income families. *Dev. Psychcol.* 30 679–689. 10.1037/0012-1649.30.5.679

[B82] Willard J. A. Agache A. Jaekel J. Glück C. W. Leyendecker B. (2015). Family factors predicting vocabulary in Turkish as a heritage language. *Appl. Psycholinguist.* 36 875–898. 10.1017/S0142716413000544

[B83] Winsler A. Kim Y. K. Richard E. R. (2014). Socio-emotional skills, behavior problems, and Spanish competence predict the acquisition of English among English language learners in poverty. *Dev. Psychol.* 50 2242–2254. 10.1037/a0037161 24911567

[B84] Yazejian N. Bryant D. Freel K. Burchinal M. (2015). High-quality early education. Age of entry and time in care differences in student outcomes for English-only and dual language learners. *Early Child Res. Q.* 32 23–39. 10.1016/j.ecresq.2015.02.002

